# Efficacy and safety of ripertamab for treating primary membranous nephropathy among adults: a multicenter, retrospective, real-world study

**DOI:** 10.3389/fimmu.2025.1540694

**Published:** 2025-03-26

**Authors:** Xichao Wang, Xinyuan Song, Na Sun, Wenxiu Chang

**Affiliations:** Department of Nephrology, Tianjin First Central Hospital, Tianjin, China

**Keywords:** primary membranous nephropathy, ripertamab, clinical remission rate, safety, real-world study

## Abstract

**Background:**

Ripertamab has been used in an off-label manner for treating primary membranous nephropathy (PMN) in real-world settings in China, despite limited evidence supporting the efficacy of this drug. This multicenter, retrospective study is the first to assess the effectiveness and safety of ripertamab for treating PMN in a real-world Chinese clinical setting.

**Methods:**

Adult patients with PMN who were treated with at least one course of ripertamab alone were included in this study. Patients were categorized into two groups based on their prior treatment of PMN: the initial therapy group and the non-initial therapy group. The primary outcome was the occurrence of complete remission (CR) or partial remission (PR) at 6 and 12 months. The secondary outcomes included the time to achieve remission, relapse rate and the incidence of adverse events (AEs).

**Results:**

Fifty-two patients were ultimately included for analysis. Among these patients, 39 received ripertamab as initial therapy, while 13 were in the non-initial therapy group. The median follow-up duration was 8.7 (4.7, 11.3) months. At 6 months, 24/40 (60.0%) patients achieved clinical remission, with 2/40 (5.0%) achieving CR and 22/40 (55.0%) achieving PR. At 12 months, 22 patients completed follow-up: 2 (9.1%) achieved CR, and 15 (68.2%) achieved PR. The median time to remission for the entire cohort was 90.5 (32, 165) days and four of the 52 patients (7.7%) relapsed. The initial therapy group had a higher remission rate at 12 months than the non-initial therapy group [13/15 (86.7%) vs. 4/7 (57.1%)]. Additionally, the initial therapy group achieved remission more quickly than the non-initial therapy group [79.0 (36, 112) vs. 165.0 (30, 313) days]. Ripertamab was well tolerated, with 9.6% (5/52) of patients experiencing AEs; none of the AEs were severe.

**Conclusion:**

Ripertamab demonstrated efficacy and good tolerability for the treatment of PMN in a Chinese real-world setting. These findings support the use of ripertamab as a therapeutic option for PMN patients and suggest the need for further investigation into its long-term safety and efficacy.

## 1 Introduction

Primary membranous nephropathy (PMN) is an autoimmune disease that is one of the most common causes of nephrotic syndrome among adults (1). In China, the prevalence of PMN is increasing annually, accounting for 23.4% of cases of nephrotic syndrome, second only to immunoglobulin A (IgA) nephropathy at 28.1% (2, 3). PMN can manifest at any age but is more prevalent among individuals aged 50 to 60 (1), with a male-to-female ratio of 2:1 (4). Although approximately one‐third of PMN patients may achieve spontaneous complete remission (CR) or partial remission (PR) after receiving conservative treatment, the remaining one-third of patients have an elevated risk of disease progression, potentially leading to end‐stage renal disease (ESRD) within 10 years (5).

Currently, the treatment approach for patients with PMN is highly individualized, with experts recommending tailored regimens based on each patient's risk evaluation. The Kidney Disease: Improving Global Outcomes (KDIGO) 2021 Clinical Practice Guideline for the Management of Glomerular Diseases recommends that all PMN patients receive optimal supportive care, along with the administration of rituximab (RTX) or cyclophosphamide (CTX) as well as glucocorticoids (GC) every other month for 6 months for patients with at least one risk factor for disease progression (6). In recent years, targeted therapy has emerged as a promising and precise treatment modality, complementing or replacing traditional immunosuppressive therapies and providing a novel avenue for personalized treatment. RTX is a monoclonal antibody that targets the CD20 antigen and specifically binds to the CD20 antigen on the surface of B lymphocytes (7). The vast majority of PMN patients who are treated with RTX achieve either CR or PR, with an overall remission rate of 60% to 80% (8, 9). Despite its favorable efficacy and safety profile, the high cost of RTX has resulted in limited accessibility in China.

Ripertamab (SCT400) is a recombinant human-mouse chimeric anti-CD20 immunoglobulin G1 (IgG1) monoclonal antibody that shares the same amino acid sequence of antigen binding sites and a variable region as RTX, with a single amino acid difference at position 219 of the heavy chain constant region 1 (10). It was approved by the National Medical Products Administration in 2022 for treating B-cell non-Hodgkin lymphoma (NHL). Previous studies have shown that ripertamab has similar pharmacokinetics, pharmacodynamics, immunogenicity, and safety profiles to RTX in Chinese patients with CD20-positive B-cell NHL (10, 11). In a real-world setting, ripertamab has also been utilized in patients with PMN; however, its efficacy and safety for treating PMN has not yet been reported.

Given the potential benefits of ripertamab, we designed this multicenter, retrospective study to evaluate the efficacy and safety of ripertamab for treating PMN among Chinese patients. This study aimed to elucidate the role of ripertamab in managing PMN, and to provide a scientific foundation for its clinical application.

## 2 Materials and methods

### 2.1 Study population

This was a multicenter, retrospective, real-world study. PMN was diagnosed through renal biopsy and exclusion of secondary causes of glomerular disease (lupus nephritis, Henoch-Schonlein purpuric nephritis, or diabetic nephropathy, etc.), based on medical history, symptoms, physical examination, serological tests and/or serum anti-phospholipase A2 receptor (anti-PLA2R) test. The inclusion criteria were as follows: (i) adult patients with biopsy-confirmed PMN; (ii) patients who received at least one course of ripertamab alone as an initial or non-initial treatment for PMN; and (iii) patients who received ripertamab between January 2023 and October 2024. The exclusion criteria were as follows: (i) patients diagnosed with secondary nephrotic syndromes such as lupus nephritis, Henoch-Schonlein purpuric nephritis, or diabetic nephropathy; (ii) patients who had received RTX or its biosimilars within three months before enrollment or during the treatment period; (iii) patients who combined use of GC or immunosuppressive agents (eg. pcalcineurin phosphatase inhibitors, alkylating agents); (iv) patients involved in other clinical trials during the treatment period; (v) patients with a history of malignant tumors or organ transplantation. Patients were categorized into two groups based on their prior treatment before ripertamab: the initial therapy group and the non-initial therapy group.

Patients were recruited from Tianjin First Central Hospital, Tianjing Fifth Central Hospital and Tianjin Dongli Hospital. The study was approved by the institutional review board of Tianjin First Central Hospital (No. 20240423-1). Data collection, storage, and analysis were conducted in accordance with the guidelines for using real-world data to generate real-world evidence, as outlined by the Center for Drug Evaluation in China.

### 2.2 Treatment and follow-up

In the real-world setting, ripertamab was administered according to two regimens: (1) intravenously at 375 mg/m² once a week for 4 weeks, or (2) 1 g per dose every two weeks for a total of 2 doses. To minimize the infusion reaction, patients received intravenous methylprednisolone (40 mg) and oral cetirizine (10 mg) before infusion. After the initial administration, the decision to reinject once or twice with 375 mg/m^2^ × 1-2 doses or 1 g/dose × 1-2 doses was based on anti-PLA2R antibody or clinical remission status at the 6-month follow-up.

Demographic characteristics, treatment information and laboratory data were obtained from the databases of each medical center for patients enrolled at the beginning of ripertamab therapy and during follow-up. The follow-up time was 1, 3, 6, 9, and 12 months, with subsequent 6-month intervals. The endpoints were ESRD, loss to follow-up or death. Laboratory parameters, including 24-h urinary protein (UP), serum albumin, serum creatinine and estimated glomerular filtration rate (eGFR), were assessed at baseline and at every visit. AEs related to ripertamab were evaluated during the infusions and throughout the entire follow-up period.

### 2.3 Outcomes

The primary outcomes were the occurrence of CR or PR at 6 and 12 months. The secondary outcomes included the time to remission, relapse rate and the incidence of AEs. Remission was defined according to the 2021 KDIGO guidelines: (1) CR was defined as a 24-h UP ≤ 0.3 g/d with normal kidney function; (2) PR was defined as a reduction in proteinuria of more than 50% from baseline with a value of 24-h UP < 3.5 g and normal kidney function. Clinical remission rate is defined as the rate of CR and PR. The time to remission was defined as the time from the first ripertamab injection to the achievement of CR or PR. Relapse was defined as reoccurrence of 24-h UP >3.5 g in patients who had achieved CR or PR. ESRD was defined as a creatinine clearance less than 15 mL/min at the last follow-up, initiation of dialysis, or renal transplantation.

### 2.4 Statistical analysis

Data analysis was performed via SAS version 9.4. Continuous variables were summarized via descriptive statistics, including means, standard deviations, medians, and interquartile ranges (IQRs). Categorical variables are presented as frequencies and percentages, with missing data excluded from percentage calculations. Baseline parameters were compared between treatment groups via the Mann–Whitney test or chi-square test as appropriate. Longitudinal differences in clinical parameters from baseline to various time points were analyzed via the Mann–Whitney test. The Kaplan−Meier method was used to compare the clinical remission of patients in different groups. The results are presented as adjusted hazard ratios with 95% confidence intervals (CIs). Statistical significance was defined as a two-sided alpha level of 0.05, with all tests being two-sided.

## 3 Results

### 3.1 Baseline characteristics

**Figure 1:**
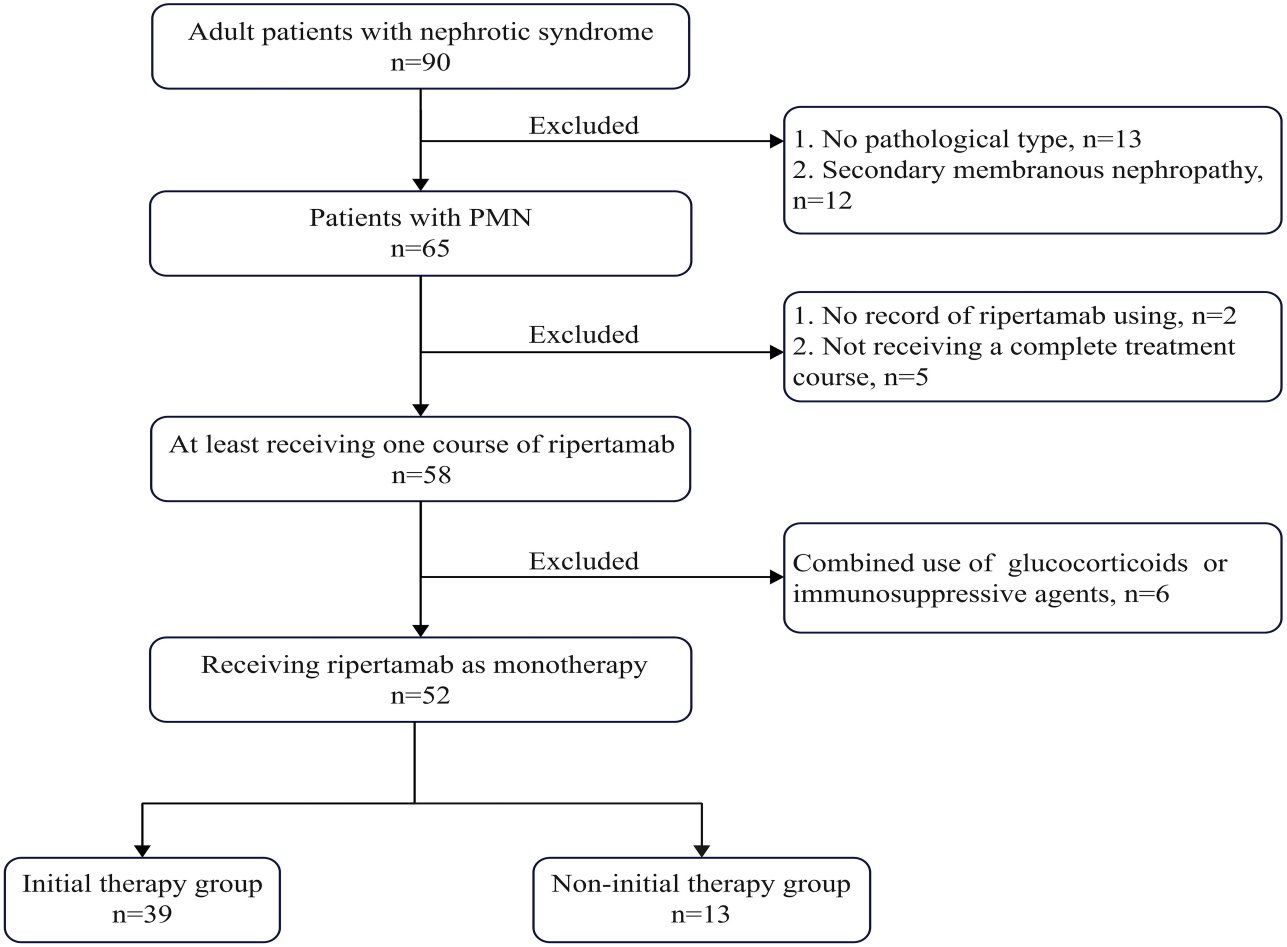
Flow chart of the patient selection process, PMN, primary membranous nephropathy.

A total of 52 adult patients with PMN were ultimately included for analysis after screening participants against the inclusion and exclusion criteria (**Figure 1**). Thirty-nine patients received ripertamab as their initial therapy, whereas 13 patients were treated with ripertamab after experiencing a relapse or failure of prior treatments.

The cohort was predominantly male (80.8%), with a median age of 52.5 (37.5, 62.5) years (**Table 1**). Most patients were nondiabetic (76.9%) and had hypertension (75.0%). The median baseline 24-h UP level was 6.5 (4.5, 9.7) g/d, the median serum albumin level was 24.5 (21.5, 30.3) g/L, the median serum creatinine level was 80.0 (66.3, 88.5) μmol/L, and the mean eGFR was 93.4 ± 24.4 mL/min/1.73m². Among the 25 patients with available anti-PLA2R levels, 18 (72.0%) tested positive. A greater proportion of patients in the initial therapy group tested positive for PLA2R antibodies (12/14, 85.7%) than in the non-initial therapy group (6/11, 54.5%), although this difference was not statistically significant (*p* = 0.227).

**Table 1:** Baseline characteristics of patients with primary membranous nephropathy receiving ripertamab.

Characteristic	Total	Initial therapy	Non-initial therapy	t/χ2	P value
	(N=52)	(N=39)	(N=13)		
Male, n (%)	42 (80.8)	32 (82.1)	10 (76.9)	0.165	0.921
Age (year)	52.5 (37.5, 62.5)	48.0 (37.0, 61.0)	59.0 (52.0, 63.0)	4.079	0.043
Diabetes, n (%)	12 (23.1)	10 (25.6)	2 (15.4)	0.578	0.749
Hypertension, n (%)	39 (75.0)	29 (74.4)	10 (76.9)	0.034	0.983
Systolic BP (mmHg)	125.0 (120.0, 130.0)	125.0 (113.0, 130.0)	125.0 (120.0, 128.0)	0.901	0.342
Diastolic BP (mmHg)	80.0 (78.0, 85.0)	80.0 (78.0, 85.0)	80.0 (80.0, 85.0)	0.103	0.749
BMI	26.0 (23.5, 28.9)	26.0 (23.5, 28.7)	25.4 (22.7, 29.3)	0.338	0.561
Anti-PLA2R antibody positivity, n (%) ^a^	18/25 (72.0)	12/14 (85.7)	6/11 (54.5)	2.968	0.227
24-h UP (g/d)	6.5 (4.5, 9.7)	7.1 (5.5, 10.5)	5.5 (3.8, 7.7)	2.990	0.084
Albumin (g/L)	24.5 (21.5, 30.3)	23.9 (20.7, 28.1)	30.9 (23.8, 34.7)	6.593	0.010
Serum creatine (μmol/L)	80.0 (66.3, 88.5)	80.3 (66.2, 90.1)	79.7 (66.4, 85.4)	0.003	0.958
eGFR (mL/min/1.73m^2^) ^b^	93.4 ± 24.4	97.1 ± 22.2	82.7 ± 27.9	1.878	0.066
Missing, n	2	2	0		
Cholesterol (mmol/L)	6.2 (4.9, 7.1)	6.4 (5.3, 7.1)	4.7 (4.2, 7.0)	2.305	0.129
Missing, n	13	6	7		
Triglycerides (g/L)	2.3 (1.5, 3.5)	2.3 (1.5, 3.7)	2.3 (2.1, 3.1)	0.002	0.968
Missing, n	13	6	7		
White blood cell (10^9^/L)	7.3 ± 1.9	7.2 ± 1.8	7.8 ± 2.5	-0.976	0.334
Missing, n	2	2	0		
Platelet (10^9^/L)	281.2 ± 74.3	287.5 ± 80.0	263.2 ± 53.5	1.015	0.315
Missing, n	2	2	0		
Total protein (g/L)	47.9 ± 9.7	45.9 ± 8.0	54.3 ± 11.7	-2.68	0.010
Missing, n	8	6	2		
Globulin (g/L)	21.0 ± 3.9	20.3 ± 3.4	23.1 ± 4.4	-2.25	0.029
Missing, n	6	5	1		
ALT (g/L)	18.0 (15.6, 23.2)	17.8 (15.2, 19.8)	21.5 (17.1, 28.3)	3.592	0.058
Missing, n	3	2	1		
AST (g/L)	15.2 (12.3, 20.4)	15.1 (12.5, 18.2)	15.6 (11.0, 29.6)	0.439	0.508
Missing, n	3	2	1		

Values are presented as numbers (%), medians (interquartile range), or means ± SD.

ALT, alanine aminotransferase; AST, aspartate transaminase; BMI, body mass index; BP, blood pressure; eGFR, estimated glomerular filtration rate; PLA2R, phospholipase A2 receptor; UP, urinary protein.

a Anti-PLA2R positivity was deﬁned by a value >20 U/ml.

b eGFR was calculated according to the Chronic Kidney Disease Epidemiology Collaboration equation.

Compared with the non-initial therapy group, the initial therapy group was younger [48.0 (37.0, 61.0) vs. 59.0 (52.0, 63.0) years, *p* = 0.043] and had lower levels of serum albumin [23.9 (20.7, 28.1) vs. 30.9 (23.8, 34.7) g/L, *p* = 0.010], total protein (45.9 ± 8.0 vs. 54.3 ± 11.7 g/L, *p* = 0.010), and globulin (20.3 ± 3.4 vs. 23.1 ± 4.4 g/L, *p* = 0.029).

### 3.2 Remission rate and time to achieve remission

**Figure 2:**
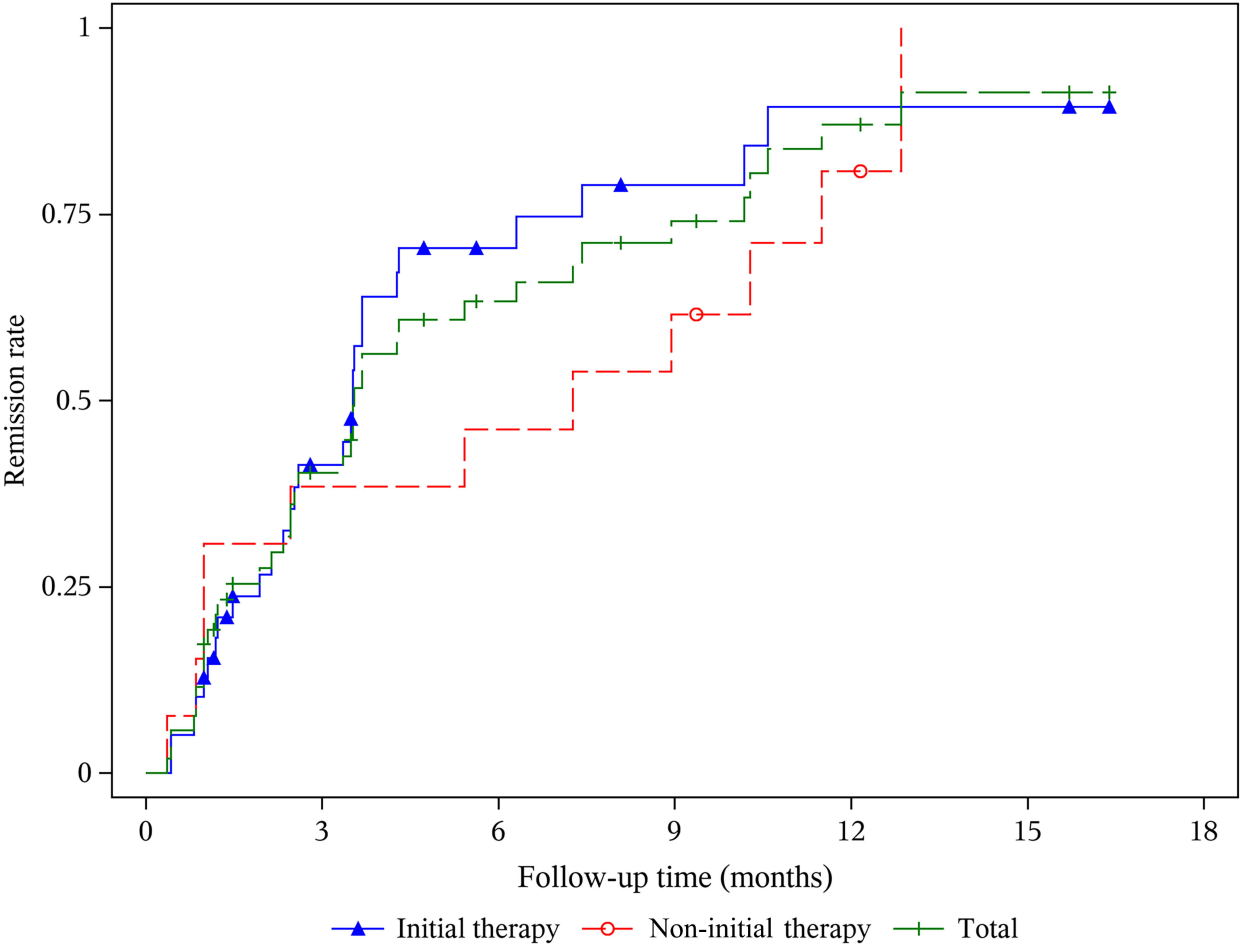
Kaplan-Meier curves for clinical remission after ripertamab in patients with primary membranous nephropathy.

The median follow-up time for the whole cohort was 8.7 (4.7, 11.3) months. At 6 months, 24/40 (60.0%) patients achieved clinical remission, with 2/40 (5.0%) achieving CR and 22/40 (55.0%) achieving PR (**Table 2**). At 12 months, 22 patients completed follow-up; 2 (9.1%) patients achieved CR, and 15 (68.2%) patients achieved PR. The median time to achieve remission across the entire population was 90.5 (32, 165) days (**Figure 2**). Notably, the initial therapy group consistently exhibited a higher total remission rate than the non-initial therapy group. Compared with the non-initial therapy group, the initial therapy group had a higher remission rate [13/15 (86.7%) vs. 4/7 (57.1%)] at 12 months and achieved remission sooner [79.0 (36, 112) vs. 165.0 (30, 313) days]. Four of the 52 (7.7%) patients relapsed with two patients in the initial therapy group and two in the non-initial therapy group, respectively.

**Table 2:** Remission rate of ripertamab in patients with primary membranous nephropathy.

Study time points	Total (n=52)	Initial therapy (n=39)	Non-initial therapy (n=13)
	Complete remission rate/ No. (%)		
3 months	0/46 (0.0)	0/34 (0.0)	0/12 (0.0)
6 months	2/40 (5.0)	2/28 (7.1)	0/12 (0.0)
9 months	3/28 (10.7)	3/18 (16.7)	0/10 (0.0)
12 months	2/22 (9.1)	2/15 (13.3)	0/7 (0.0)
	Partial remission rate/ No. (%)		
3 months	25/46 (54.3)	21/34 (61.8)	4/12 (33.3)
6 months	22/40 (55.0)	17/28 (60.7)	5/12 (41.7)
9 months	15/28 (53.6)	10/18 (55.6)	5/10 (50.0)
12 months	15/22 (68.2)	11/15 (73.3)	4/7 (57.1)
	Clinical remission rate/ No. (%)		
3 months	25/46 (54.3)	21/34 (61.8)	4/12 (33.3)
6 months	24/40 (60.0)	19/28 (67.9)	5/12 (41.7)
9 months	18/28 (64.3)	13/18 (72.2)	5/10 (50.0)
12 months	17/22 (77.3)	13/15 (86.7)	4/7 (57.1)
Time to remission (day), median	90.5 (32, 165)	79.0 (36, 112)	165.0 (30, 313)

### 3.3 Changes in laboratory indices

**Figure 3:**
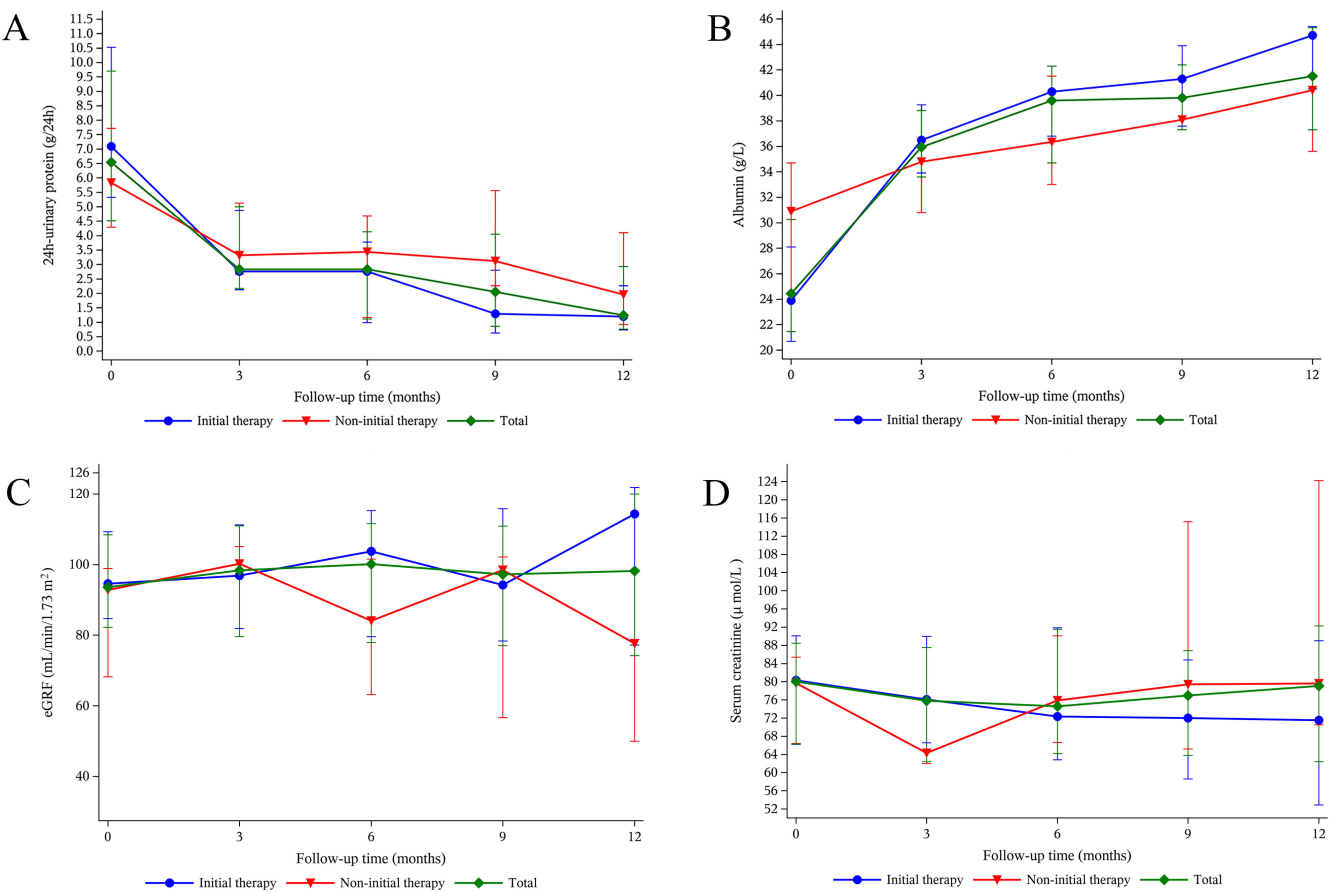
Change in levels of 24-h urinary protein **(A)**, serum albumin **(B)**, eGFR **(C)**, and serum creatinine **(D)** after ripertamab treatment. Data are presented as the medians (interquartile range) over time. eGFR: estimated glomerular filtration rate.

All PMN patients exhibited a statistically significant reduction in 24-h UP levels for the entire observation period [from a median 24-h UP level of 6.5 (4.5, 9.7) to 1.2 (0.8, 2.9) g at 12 months, *p* < 0.01] (**Figure 3A**). The observed trend of decreased 24-h UP in the initial therapy group was consistent with the trends observed in the total cohort, whereas the non-initial therapy group also showed a decreasing trend without statistical significance. In addition, the serum albumin levels significantly increased [from a median albumin level of 24.5 (21.5, 30.3) to 41.5 (37.3, 45.3) g/L at 12 months, *p* < 0.01] (**Figure 3B**). This increase was observed in both the initial and non-initial therapy groups, with statistically significant changes in both groups. Following treatment with ripertamab, the eGFR remained stable across all groups (**Figure 3C**). While the serum creatinine levels decreased in all the groups, these changes did not reach statistical significance (**Figure 3D**).

### 3.4 Adverse events

Ripertamab was well tolerated in almost all patients, with 9.6% (5/52) of patients reporting AEs. Among the reported AEs (**Table 3**), two patients experienced mild dermatological reactions, including rash and facial flushing. Additionally, one patient presented with fever and the detection of a pulmonary nodule, underscoring the need for continued monitoring of patients during and after treatment.

**Table 3:** Adverse events associated with ripertamab during follow-up.

Adverse events	Patients (n=52)
Fever	1
Rash	2
Infusion-related reactions	1
Facial flushing	2
Pulmonary nodule	1

## 4 Discussion

Ripertamab has been used in an off-label manner to manage PMN within clinical settings despite the absence of robust evidence to substantiate its use. This multicenter, retrospective study is the first to examine the effectiveness and safety of ripertamab as a treatment for PMN in the context of Chinese real-world practice. We enrolled a total of 52 PMN patients whose demographic characteristics closely aligned with those reported in previous studies (12–14), thereby providing a certain level of representativeness despite the modest sample size.

Our results indicated that ripertamab was effective in the initial therapy group, with remission rates of 67.9% at 6 months and 86.7% at 12 months. These outcomes are superior to those reported in earlier randomized controlled trials (RCTs). For example, in the MENTOR trial (15), the remission rates of RTX were 35% at 6 months and 60% at 12 months, whereas the RI-CYCLO trial (16) reported remission rates of 51% at 6 months and 62% at 12 months. Our findings are more consistent with those from Chinese retrospective studies, such as those by Wang et al. (67.5% remission at 6 months and 87.5% at 12 months of RTX) (12) and Zhang et al. (48.1% at 6 months and 80.2% at 12 months of RTX) (14). Several factors may explain why our results are superior to those of the RCTs but align with those of the Chinese retrospective studies. First, the RCTs had stricter inclusion criteria, often requiring that 24-h UP levels be measured multiple times before enrollment. Second, patients in RCTs tended to have higher baseline 24-h UP levels, which could make it more challenging to achieve remission. Third, differences in body surface area exist, as Chinese patients generally had lower body mass index and smaller body surface areas than Western patients. Consequently, patients with smaller body surface areas may receive relatively greater drug exposure, potentially leading to superior outcomes.

Compared with the initial therapy group, the non-initial therapy group had a lower remission rate after ripertamab treatment: 41.7% at 6 months and 57.1% at 12 months. In the non-initial therapy group, most patients had previously responded to immunosuppressive therapies (Supplementary material). Therefore, it can be inferred that the low response rate to ripertamab is not attributable to prior treatment failures. Our results are consistent with previous findings related to RTX, such as those reported by Zhang et al. (40% remission at 6 months) (14). A cohort study from Peking University First Hospital also revealed that 41.7% of patients achieved remission with RTX after a median follow-up of 12 months (17). Additionally, a long-term cohort study revealed that 56.3% of patients achieved remission after receiving RTX as second-line therapy, with a median follow-up of 29 months (18). In general, our 12-month remission rate is consistent with those reported in other studies, which supports the efficacy of ripertamab in this context.

With respect to CR, our study revealed that 5.0% of patients achieved CR at 6 months, and 9.1% achieved CR at 12 months. These rates are lower than those reported in the RI-CYCLO trial (8% at 6 months, 16% at 12 months) (16), the study by Zhang et al. (6.2% at 6 months, 25.9% at 12 months) (14) and the study by Wang et al. (5.0% at 6 months, 22.5% at 12 months) (12). The lower CR rate at 12 months in our cohort could be attributed to several factors. First, loss to follow-up: In real-world settings, patients who achieve CR may be less motivated to return for follow-up visits, leading to underreporting of CR rates. Only one of the six patients who achieved CR continued attending follow-up visits, which may explain the observed lower CR rate at 12 months. Second, short follow-up duration: A shorter follow-up period may also limit the detection of CR (19). For example, Dahan et al. suggested that the likelihood of achieving CR increases with longer follow-up (20). In the MENTOR trial (15), although the CR rate was 35% at 6 months, after an extended follow-up period of 17 months, 64.9% of patients treated with RTX were in remission. In our study, 57.7% (30/52) of patients did not complete the 12-month follow-up, further complicating the assessment of remission. Third, differences in treatment modality: In RCTs, patients are typically retreated with RTX at the 6-month mark, regardless of their clinical status (7, 15). In contrast, Chinese clinical practices often rely on additional RTX infusions only when B-cell counts exceed 5 cells/mL or when anti-PLA2R antibody levels remain elevated (12, 21). However, owing to constraints in testing and associated costs, monitoring of B-cell counts and anti-PLA2R antibodies is not routinely conducted in real-world settings, which may contribute to less frequent retreatment and lower CR rates than those reported in RCTs.

A previous meta-analysis demonstrated a statistically significant reduction in 24-h UP and an increase in serum albumin levels post-RTX treatment, with changes recorded at −4.90 g/d and 10.4 g/L, respectively (22). Severe proteinuria is a risk factor for the progression of PMN to ESRD, and urine protein levels are valuable for informing treatment-related decisions (23). While the changes in the serum creatinine level and eGFR were not significant, our findings align with these results, revealing a significant decrease in 24-h UP by 4.16 g/d and an increase in the serum albumin level by 16.3 g/L at 12 months. Similarly, Fiorentino et al. reported significant increases in albumin levels following RTX therapy throughout follow-up (24), a phenomenon correlated with stable renal function, which is consistent with outcomes from other studies (18, 25).

In terms of safety, ripertamab was generally well tolerated, with no major safety concerns reported. The incidence of AEs in our study was lower than that reported in previous RTX-related studies, which reported rates ranging from 22% to 80% (12–15). This discrepancy may stem from the nature of real-world studies, where some minor or nonurgent AEs might not be documented. Notably, infusion reactions, which are the most common type of AEs reported in previous studies, were relatively infrequent in our cohort. This may be attributed to the prophylactic use of antiallergic medications prior to infusion and a carefully controlled infusion rate aimed at mitigating infusion-related events. However, we cannot entirely rule out the possibility that some AEs were undetected due to the limited sample size and short duration of follow-up. Overall, the favorable safety profile of ripertamab supports its use as a viable therapeutic option for this patient population.

The treatment of PMN typically involves the combination of GC with an immunosuppressive agent, such as CTX, tacrolimus or RTX. A meta-analysis suggested that the combination of GC and CTX remained one of the most effective treatment regimens, with a remission rate of 77.4% (26). This approach is specifically preferred for patients with renal insufficiency or those with renal vascular lesions (26, 27). Additionally, tacrolimus alone or when in combination with GC, can also lead to remission of PMN. Recent studies showed that tacrolimus combined with a small dose of GC demonstrated a favorable therapeutic effects, with a remission rate of 86.7% at 6 months, but with fewer side effects (28). Our study using ripertamab to treat PMN has also yielded comparable therapeutic outcomes with minimal AEs. As previously mentioned, RTX has been proven to be effective in the treatment of PMN, and recent studies suggested that RTX treatment in patients with HCV infection does not exacerbate its infection, thus safe for such patients (29). Additionally, Zhu et al. reported that RTX combined with traditional Chinese medicine can further aid in the remission of nephropathy (30). Our study’s findings show that ripertamab has a comparable effect to RTX in treating PMN. However, whether it has the same effects in the aspects mentioned above remains to be confirmed by further studies.

Ripertamab specifically binds to B cell surface CD20 antigen, and effectively eliminates CD20-positive B-cell through complement-dependent cytotoxicity, antibody-dependent cell-mediated cytotoxicity and direct growth inhibition (31). The molecular mechanisms underlying the efficacy of ripertamab in PMN may involve multifaceted immunomodulatory pathways targeting B-cell-driven autoimmunity. Through B-cell depletion, anti-CD20 monoclonal antibody could directly reduces the production of pathogenic autoantibodies against podocyte antigens such as PLA2R and thrombospondin type-1 domain-containing 7A (THSD7A) (32, 33). This depletion extends beyond circulating B cells to include tissue-resident B cells and short-lived plasmablasts within the kidney, thereby attenuating immune complex deposition in the glomerular subepithelial space (34, 35). Additionally, anti-CD20 therapy disrupts B-cell-mediated antigen presentation to T cells, impairing the activation of autoreactive T helper cells (like the T helper cell 17) and promoting regulatory T-cell expansion, which collectively restore immune tolerance (36, 37). Complement system modulation is another critical mechanism: B-cell depletion reduces circulating levels of C5b-9 membrane attack complexes, thereby mitigating podocyte injury and preserving nephrin expression (38, 39).

This study had several limitations that should be acknowledged. The retrospective nature of this study introduces selection bias. The primary limitations include the relatively small sample size and short duration of follow-up. Furthermore, the potential for bias inherent in a single-arm study focused on a specific treatment option. Owing to constraints in testing and associated costs, critical indicators such as PLA2R antibody levels and B lymphocyte counts could not be consistently monitored, which hampers statistical analysis and diminishes the study's overall credibility. In addition, Chinese patients with kidney disease are often treated with Chinese medicines, and the inability to access this information makes it impossible to assess the impact of Chinese medicines on the efficacy of ripertamab. Despite these limitations, our study contributes valuable insights into the disease landscape and the application of ripertamab in treating PMN.

The results of this real-world study revealed that ripertamab achieved a high remission rate in patients with PMN, and the initial therapy group had a higher remission rate than did the non-initial therapy group and achieved remission sooner. Ripertamab notably reduced 24-h UP levels and increased serum albumin level. Additionally, ripertamab was generally well tolerated, with a low incidence of AEs. These findings suggest that ripertamab offers significant clinical benefits for patients with PMN, positioning it as a recommended treatment option in this patient cohort. However, as this was a retrospective study, further prospective, large-sample studies should be performed to evaluate the long-term effectiveness and safety of ripertamab for the treatment of PMN. Further research will help solidify the role of this drug in the management of PMN and enhance our understanding of its potential as a therapeutic alternative.

## Data Availability

The raw data supporting the conclusions of this article will be made available by the authors, without undue reservation.
