# Supplementary material for: Efficacy and safety of ripertamab for treating primary membranous nephropathy among adults: a multicenter, retrospective, real-world study
Source: Front Immunol. 2025 Mar 26;16:1540694. doi: 10.3389/fimmu.2025.1540694 (PMC11979284; doi:10.3389/fimmu.2025.1540694)
Supplement: Supplementary file 1 [file Table1.docx]

Supplementary Material

Table 1. The treatment information of patients in non-initial therapy group

| Interval between last prescription for primary membranous nephropathy and initiation of ripertamab (month) | Number of patients | Drug | Outcome |
| --- | --- | --- | --- |
| More than 12 months | 10 | Cyclophosphamide, Tacrolimus, Mizoribine, Glucocorticoids | Five patients was CR, three patients was PR, and two patients’ efficacy data were missing. |
| 6-12 months | 2 | Tacrolimus, Mizoribine | One patients was CR and one was PR. |
| 3-6 months | 1 | Glucocorticoids | Non-response |

CR: complete remission; PR: partial remission
